# Antibiotic resistance gene-free probiont administration to tilapia for growth performance and *Streptococcus agalactiae* resistance

**DOI:** 10.14202/vetworld.2023.2504-2514

**Published:** 2023-12-25

**Authors:** Mira Mawardi, Agustin Indrawati, Angela Mariana Lusiastuti, I Wayan Teguh Wibawan

**Affiliations:** 1 Division of Medical Microbiology, School of Veterinary Medicine and Biomedical Sciences, IPB University, Jl. Agatis Kampus IPB Dramaga Bogor, Jawa Barat, 16680 Indonesia; 2 Government of Indonesia Ministry of Marine Affairs and Fisheries, Main Center for Freshwater Aquaculture - Ministry of Marine Affairs and Fisheries, Jl. Selabintana No. 37, Selabatu, Kec. Cikole, Kota Sukabumi, Jawa Barat 43114, Indonesia; 3 Research Center for Veterinary Sciences. National Research and Innovation Agency, KST BRIN Soekarno Cibinong Bogor, 16911, Jawa Barat, Indonesia

**Keywords:** *Bacterium*, *Lactococcus garvieae*, *Priestia megaterium*, Probiotic, *Streptococcus agalactiae*, Tilapia

## Abstract

**Background and Aim:**

The rapid development of aquaculture as a major food sector is accompanied by challenges, including diseases that affect tilapia farming worldwide. One such infectious disease caused by *Streptococcus agalactiae* poses a serious threat to tilapia populations. Probiotics have emerged as a potentially safe preventive measure against *S. agalactiae* infection. However, antimicrobial resistance from antibiotic-resistant bacteria remains a concern because it can lead to the spread of resistant bacteria and serve as a reservoir of antibiotic-resistant genes in fishes and the surrounding environment. This study aimed to identify candidate probiotic bacteria capable of promoting tilapia growth, providing resistance to *S. agalactiae* infection, devoid of potential pathogenicity, and free from antibiotic resistance genes. Subsequently, the performance of these probiotic candidates in tilapia was evaluated.

**Materials and Methods:**

*Lactococcus garvieae, Priestia megaterium, Bacterium* spp.*, Bacillus megaterium, Bacillus subtilis*, and *Bacillus pumilus* were examined to assess their antibacterial properties, hemolytic patterns, and antibiotic resistance genes. We used the specific primers *tetA*, *tetB*, *tetD*, *tetE*, *tetO*, *tetQ*, *ermB*, and *qnrS* that were used for antibiotic resistance gene detection. *In vivo* probiotic efficacy was evaluated by administering probiotic candidates in tilapia feed at a concentration of 1 × 10^6^ colonies/mL/50 g of feed over a 60-day maintenance period. Resistance to *S. agalactiae* infection was observed for 14 days after the challenge test.

**Results:**

*Lactococcus garvieae*, *P. megaterium*, and *Bacterium* spp. were identified as promising probiotic candidates among the bacterial isolates. On the other hand, *B. megaterium*, *B. subtilis*, and *B. pumilus* carried resistance genes and exhibited a β hemolytic pattern, rendering them unsuitable as probiotic candidates. The selected probiotic candidates (*L. garvieae*, *P. megaterium*, and *Bacterium* spp.) demonstrated the potential to enhance tilapia growth, exhibited no pathogenic tendencies, and were free from antibiotic resistance genes. Supplementation with *L. garvieae* and *Bacterium* spp. enhanced tilapia resistance to *S. agalactiae* infection, whereas *P. megaterium* supplementation showed an insignificant survival rate compared with controls after the challenge test period.

**Conclusion:**

Probiotics, particularly *L. garvieae, P. megaterium*, and *Bacterium* spp., enhance growth and resistance against *S. agalactiae* infection, without harboring antibiotic resistance genes. Selecting probiotic candidates based on antibiotic resistance genes is essential to ensure the safety of fish, the environment, and human health.

## Introduction

Aquaculture plays a key role as a significant source of food protein for human consumption and fisheries farming effectively fulfills around 20% of global human protein requirements. Tilapia is a prominent fishery commodity worldwide [1, 2], and its importance extends to Indonesia’s fisheries sector [3]. Indonesia’s fishery production continues to witness steady growth, catering to both domestic and export demands, in response to growing demand. The cultivation of Tilapia encounters diverse challenges, with disease problems being a recurrent obstacle [4]. Diseases caused by *S. agalactiae* infection are frequently observed and can result in significant economic losses [5, 6]. Streptococcosis is attributed to this bacterium and is commonly found in small and consumed fish [7]. Bacterial contribution to the cultivation environment dominates over other groups of microorganisms, significantly influencing fish health [8]. As a result, significant efforts have been made to prevent and control infectious diseases in cultivated fish, including the strategic use of antibiotics, probiotics, immunostimulants, and vaccines.

Probiotics, which generally consist of beneficial bacteria with positive effects on the host or the environment, have been extensively studied and reviewed in the field of aquaculture [9–14]. These probiotics are commonly supplemented through feed to enhance health, growth, and endurance in fish [15–20], increase enzyme activity, and regulate environmental quality [21–26]. Probiotics have shown resistance to environmental stress [27] and positively influence gut microbial activity, physiological regulation, and structural morphology in fish [28]. In addition, probiotics can produce bacteriocins, which help maintain a balanced microbial consortium in the fish digestive tract [29, 30]. Moreover, probiotics have the ability to inhibit or eliminate the growth of pathogenic bacteria in fish [31–36]. In addition to probiotics, antimicrobials are commonly used in aquaculture to treat and prevent infections. However, the use of antimicrobials may lead to the development of antimicrobial resistance, posing a serious threat to the health of aquatic ecosystems [37, 38]. The presence of virulent and antibiotic-resistant pathogenic bacteria has also been reported in research reviews. Antimicrobial resistance in microorganisms continues to increase and spread rapidly in the aquaculture sector [39–42]. Antimicrobial-resistant bacteria have been extensively reported in various aspects of aquaculture, including fish, sediment, and water. Moreover, the relationship between fish and their environment has been investigated in species [43, 44] such as channel catfish, *Ictalurus punctatus* [45], *Alburnus alburnus* [46], sea bass [47], yellow tail fish [48], common carp and koi carp [49], shrimp [50], Ebro barbel (*Luciobarbus graellsii*), brown trout (Salmo trutta) [51], and tilapia (*Oreochomis niloticus*) [52, 53]. Other sectors, such as animal husbandry and household waste, influence resistance genes in aquaculture [54–66]. The interconnected roles of different sectors can impact the quality of the environment and contribute to the distribution of microorganisms, including the spread of antibiotic-resistant bacteria. Resistant microorganisms can be spread from humans, animals, and the environment [67, 68]. According to Odell *et al*. [69], resistant microorganisms in the aquatic environment can affect aquatic animals and terrestrial ecosystems. Considering these findings, detecting resistance genes in probiotic candidate bacteria is imperative for their effective use in aquaculture practices.

The novelty of this research is to obtain probiotic bacteria devoid of resistance genes and to analyze their efficacy in tilapia to enhance productivity while ensuring safety for both fish and the environment. The selection of bacteria as probiotic candidates requires careful consideration of the presence of antibiotic-resistant genes. To date, no studies have documented the selection of probiotic candidates through a comprehensive assessment of their enzymatic, antibacterial, and hemolytic capabilities, along with the identification of antibiotic--resistance genes, followed by evaluating their performance in tilapia.

This study aimed to identify candidate probiotic bacteria capable of promoting tilapia growth, providing resistance to *S. agalactiae* infection, devoid of potential pathogenicity, and free from antibiotic resistance genes. Subsequently, the performance of these probiotic candidates in tilapia was evaluated.

## Materials and Methods

### Ethics approval

This study was approved by the Animal Code of Ethics Committee at the Faculty of Veterinary Medicine, Bogor Agricultural Institute, IPB University, under protocol number: 023/KEH/SKE/VIII/2022.

### Study period and location

This study was conducted from January to June 2023 at the Center for Freshwater Aquaculture Fisheries in Sukabumi and the Medical Microbiology Laboratory, School of Veterinary and Biomedical Medicine at the University of IPB.

### Study design

The bacterial isolates used in this study, including *L. garvieae, P. megaterium, Bacterium, B. megaterium, B. subtilis*, and *B. pumilus*, were obtained from previous study of Mawardi *et al*. [70]. In this research phase, an evaluation of antibacterial activity, hemolytic activity, and detection of antibiotic resistance genes was performed, which had not been performed in previous investigations.

### Anti-streptococcal activity

Probiotic candidates for antibacterial activity against *S. agalactiae* were selected using the disk diffusion method. The bacteria were cultured on Nutrient Broth medium (Oxoid, France) at 28°C for 24 h. *Streptococcus agalactiae* bacteria were inoculated using a sterile cotton swab on Muller Hilton Agar medium (MHA, Sigma, USA). Disc paper without antibiotics (Oxoid) was placed on MHA media previously inoculated with *S. agalactiae* bacteria. Ten microliters of the probiotic candidate bacterial suspension were placed on paper disks and incubated at 28°C for 24 h. Inhibition zones formed around the bacteria were measured using an electronic digital caliper (mm). Antibacterial activity tests and genetic characterization of *L. garvieae* were performed (data not shown). Inhibition zone testing of *S. agalactiae* was performed as a control using oxytetracycline, enrofloxacin, and erythromycin disks (Oxoid).

### Hemolytic pattern activity

Blood agar (Oxoid) and 5% sheep blood supplement were used to identify the hemolytic pattern. Bacteria were inoculated into the media and incubated at 28°C for 24 h. Bacteria that were not present and showed a zone of inhibition (hemolytic) were selected as probiotic candidates.

### Characterization of antibiotic resistance genes

Identification of antibiotic-resistance genes in bacterial isolates was conducted using molecular methods with specific primers (Table-1) [71–74]. The isolates used in this study have not been previously tested for resistance genes. DNA was extracted from a single colony using a DNeasy^®^ blood and tissue kit (Qiagen, Germany). DNA extraction results were subjected to amplification using a GoTaq^®^Green premix kit (Promega, USA). The polymerase chain reaction (PCR) amplification mixture comprised 2 μL of sample DNA, 12.5 μL of GoTaq^®^ Green, 1 μL of forward primer, and 1 μL of reverse primer pair (Primer Integrated DNA Technologies, Singapore), 8.5 μL of ddH_2_O with a final primary concentration of 0.4 μM in a 25 μL amplification volume. The PCR program consisted of initial denaturation at 95°C, followed by 40 cycles of denaturation at 95°C for 30 s, annealing at 30 s (Table-1), and elongation at 72°C for 2 min. A final extension step was performed at 72°C for 5 min.

**Table-1:** Characterization of antibiotic resistance genes using primary designs.

Target Genes	Sequences (5’- 3’) (FW-RV)	Annealing temperatures (ºC, T_A_)	Amplicon size (bp)	Reference
*tet*A	GCT ACA TCC TGC TTG CCT TC CAT AGA TCG CCG TGA AGA GG	60	210	[71]
*tet*B	TAC GTG AAT TTA TTG CTT CGG ATA CAG CAT CCA AAG CGC AC	58	206	[72]
*tet*D	AAA CCA TTA CGG CAT TCT GC GAC CGG ATA CAC CAT CCA TC	63	787	[71]
*tet*E	GGT ATT ACG GGA GTT TGT TGG AAT ACA ACA CCC ACA CTA CGC	61	199	[72]
*tet*O	ACG GAR AGT TTA TTG TAT ACC TGG CGT ATC TAT AAT GTT GAC	62	171	[72]
*tet*Q	TTA TAC TTC CTC CGG CAT CG ATC GGT TCG AGA ATG TCC AC	61	904	[71]
*erm*B	GAA AAG GTA CTC AAC CAA ATA AGT AAC GGT ACT TAA ATT GTT TAC	55	639	[73]
*qnr*S	ACG ACA TTC GTC AAC TGC AA TAA ATT GGC ACC CTG TAG GC	55	417	[74]

### Evaluation of the efficacy of probiotic bacteria through feeding supplementation and challenge trials

The efficacy of probiotics was evaluated on a laboratory scale to determine the efficiency of growth and resistance to *S. agalactiae* infection in tilapia *in vivo*. *Lactococcus garvieae*, *P. megaterium*, and *Bacterium* spp. were the probiotic bacteria used for the *in vivo* tests. We tested the efficacy of probiotics after treatment on tilapia, including analysis of growth, hematology, and non-specific immune responses.

### Preparation of the feed

For the *in vivo* test, the commercial feed was mixed with a suspension of probiotic bacteria containing 1 × 10^6^ colonies/mL/50 g of feed and administered for 60 days. The concentrations of probiotic bacteria were determined using the total plate count method with plate count agar (PCA; Oxoid). The study design incorporated commercial feed mixed with *L. garvieae*, *P. megaterium*, and *Bacterium*, all of which exhibited the hemolytic pattern and demonstrated no antibiotic resistance genes.

### Experimental fish preparation

Tilapia was obtained from the Center for Freshwater Aquaculture Fisheries, Sukabumi, Indonesia, with an average weight of 14.39 ± 1.02 g and a density of 20 fish per container. The fish was acclimatized for 3 weeks before the trial to assess their health status. During the acclimatization period, the fish exhibited no signs of illness, and no mortality was observed. Following acclimatization, the fish was fed the experimental feed twice daily at 09.00 AM and 16.00 PM for 60 days at a dose of 5% of the biomass weight. Table-2 summarizes the experimental design for evaluating the efficacy of probiotic bacteria through feed.

**Table-2:** Experimental design of the efficacy of probiotic bacteria through feed.

Treatments	Detail
1	*L. garvieae*
2	*P. megaterium*
3	*Bacterium* spp.
4	Control without probiotic addition, with challenge test
5	Control without probiotic addition, without a challenge test

### Growth performance analysis

Data on fish weight and feed consumption were collected at the beginning and end of the experiment to analyze the growth performance of tilapia, including the total weight gain (WG, %), specific growth rate (SGR), feed conversion ratio (FCR), and survival rate (SR, %).

Weight gain = Final weight (g) − Initial weight (g) × 100

Survival rate = Final number of fish/initial number of fish × 100

Feed conversion ratio = Total feed consumption (g)/(final weight [g] − Initial weight [g]) × 100

Specific growth rate = (In[final wight] In[initial wight])/days] × 100.

### Challenge test

As a control group, fish that had received the experimental feed without probiotics was subjected to a challenge test using *S. agalactiae* at a concentration of 1 × 10^6^ colonies/mL through intramuscular injection of 0.1 mL/fish. The fish mortality rate was observed during the 2-week challenge test period, and the SR was calculated.

### Hematology parameters

Hematological analysis was performed on fish samples before and after the challenge test, measuring the total leukocyte count and hematocrit percentage [75].

### Parameters of non-specific immunity

Non-specific immunity parameters include phagocytic activity and respiratory burst activity [76]. The lysozyme activity was evaluated before and after the challenge test [77].

### Statistical analysis

Statistical Package for the Social Sciences software version 26 (IBM Corp., NY, USA) was used for data analysis. Levene’s test was conducted to confirm the homogeneity of variance level, whereas the Shapiro–Wilk test was used to assess data normality. One-way analysis of variance was employed to test differences between experimental groups for data with normal distribution and homogeneous variance. The Kruskal–Wallis analysis was used to test differences if the data were not normally distributed and/or the variance was not homogeneous. Significant differences (p = 0.05) between treatment groups were analyzed using Tukey’s test. Further testing was conducted using the Mann–Whitney U test in cases where the Kruskal–Wallis test was applied.

## Results

### Anti-streptococcal activity

**Figure-1:**
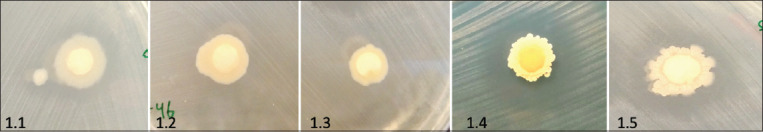
The antibacterial activity of the isolates is illustrated by the presence of an inhibition zone around the bacteria.

The antibacterial activity against *S. agalactiae* was evaluated using the disk diffusion method. The results indicated that *Bacterium* spp. (1.1), *B. pumilus* (1.4), and *B. subtilis* (1.5) exhibited inhibitory effects on the growth of *S. agalactiae*, as evidenced by the formation of a distinct inhibition zone around these bacterial isolates. Conversely, *P. megaterium* (1.2) and *B. Megaterium* (1.3) isolates demonstrated any inhibition zones around the bacteria (Figure-1). The size of the inhibition zones varied among the tested isolates, as shown in Table-3. The inhibition zones of *S. agalactiae* when treated with the tetracycline, oxytetracycline, enrofloxacin, and erythromycin were 32.22 ± 1.21 mm, 24.00 ± 0.00 mm, 19.07 ± 2.31 mm, and 25.10 ± 1.50 mm, respectively.

**Table-3:** Bacterial isolates showing anti-*Streptococcus agalactiae*, hemolytic patterns, identification based on 16S rDNA sequences and detection of antibiotic resistance genes. *Antibacterial activity was carried out by co-culture method (data are not shown) - Negative; + Positive.

Isolate/ parameter	*L. garvieae*	*P. megaterium*	*Bacterium*	*B. megaterium*	*B. pumilus*	*B. subtilis*
Zone diameter of (mm) anti-Streptoccocus	*	6.00 ± 0.00	22.50 ± 4.17	6.00 ± 0.00	18.50 ± 0.02	19.34 ± 0.36
Hemolytic pattern	γ	γ	γ	γ	β	γ
Antibiotic gene targets:						
*tet*A	-	-	-	-	+	+
*tet*B	-	-	-	+	-	-
*tet*E	-	-	-	-	-	-
*tet*D	-	-	-	-	-	-
*tet*O	-	-	-	-	-	-
*tet*Q	-	-	-	-	-	-
*erm*B	-	-	-	-	-	-
*qnr*S	-	-	-	-	-	-

### Hemolytic pattern activity

**Figure-2:**
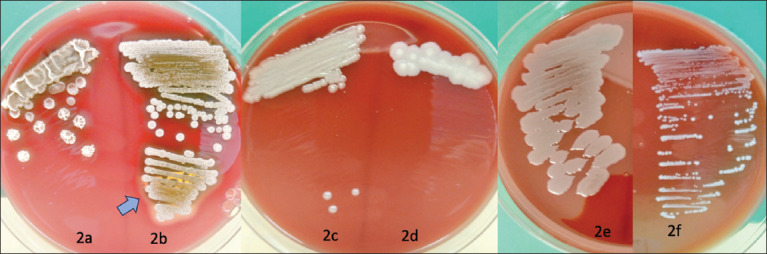
Hemolytic pattern of isolates on blood agar media supplemented with 5% sheep blood. The following isolates were examined (a) *Bacillus subtilis*, (b) *Bacillus pumilus*, (c) *Bacillus megaterium*, (d) *Priestia megaterium*, (e) *Bacterium* spp., and (f) *Lactococcus garvieae*. Notably, *Bacillus pumilus* displayed the presence of an inhibition zone around the bacteria, indicated by the blue arrow.

Blood agar (Oxoid) supplemented with 5% sheep blood was used to assess the hemolytic pattern. Our results revealed that certain isolates exhibited a hemolytic pattern designated as “hemolytic γ” and were considered probiotic candidates, as indicated by the absence of an inhibition zone around bacteria. *Bacillus subtilis* (4a), *B. megaterium* (4c), *P. megaterium* (4d), *Bacterium* (4e), and *L. garvieae* (4f) were isolated. On the other hand, *B. pumilus* (4b) demonstrated a zone around the characteristic “hemolytic β pattern” (Figure-2).

### Characterization of antibiotic resistance genes

**Figure-3:**
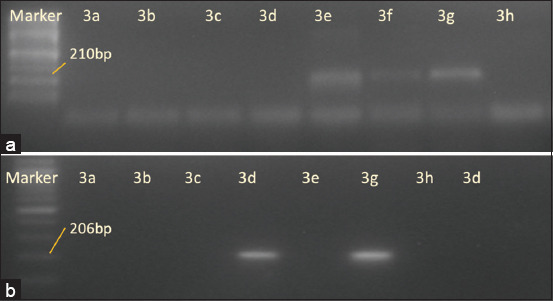
The electrophoretic profile detection of the *tetA* (above) and *tetB* (bottom) antibiotic-resistant genes. (a) *Bacterium* spp., (b) *Lactococcus garvieae*, (c) *Bacillus firmus*, (d) *Bacillus megaterium*, (e and f) *Bacillus subtilis*, (g) positive control, and (h) negative control.

Isolates considered probiotic candidates were selected based on the absence of antibiotic-resistance genes. Detection of antibiotic resistance genes was performed using molecular methods. Bacterium, *L. garvieae*, and *P. megaterium* were negative for the presence of antibiotic resistance genes. However, *B. pumilus* and *B. subtilis* isolates were positive for *tetA* and *tetB. megaterium* isolate was positive for *tetB* (Figure-3). Detecting antibiotic resistance genes *tetE*, *tetD*, *tetO*, *tetQ*, *ermB*, and *qnrS* yielded negative results for all isolates (Table-3).

*Lactococcus* *garvieae*, *P. megaterium*, and *Bacterium* spp. fulfilled the criteria as probiotic candidates, showing hemolytic patterns and no detection of antibiotic resistance genes (*tetA*, *tetB*, *tetE*, *tetD*, *tetO*, *tetQ*, *ermB*, and *qnrS*) (Table-3). These isolates have been selected for further *in vivo* performance testing on tilapia.

Growth performance, hematological features, non-specific immunity, and resistance to *S. agalactiae*. The efficacy of probiotic bacteria was evaluated before and after the challenge test using various parameters, including growth performance, hematological parameters, and the nonspecific immune system of tilapia.

As shown in Table-4, the growth data, represented by WG during the experiment, and SGR exhibited variations among the treatments. Notably, Bacterium spp. showed the highest value. In addition, the FCR of *Bacterium* spp. treatment was lower than the other treatments. The total leukocyte and hematocrit values did not show significant differences in tilapia before the challenge test. Regarding the non-specific immune system parameters, respiratory burst activity and lysozyme activity were significantly higher in fish that received treatment 3 compared with control fish that received treatment 5. However, the phagocytic activity values did not significantly differ.

**Table-4:** Growth performance, hematological parameters, and non-specific immunity in tilapia after 60 days of trial feeding.

Parameters	Treatments				

	*L. garvieae*	*P. megaterium*	*Bacterium* spp.	Control without probiotic addition, with challenge test	Control without probiotic addition, without a challenge test
SR (%)	100%	100%	100%	100%	100%
WG (%)	206.28 ± 7.85^bc^	215.45 ± 2.39^c^	221.6 ± 9.57^c^	184.07 ± 6.22^a^	190.89 ± 4.06^ab^
FCR	1.03 ± 0.01^b^	1 ± 0.02^ab^	0.96 ± 0.01^a^	1.46 ± 0.02^d^	1.42 ± 0.01^c^
SGR (%/day)	1.87 ± 0.05^bc^	1.91 ± 0.01^c^	1.95 ± 0.05^c^	1.74 ± 0.04^a^	1.78 ± 0.03^ab^
TL (cell.mm)^(-3)	23583.33 ± 1086.66	23800 ± 3740.32	21200 ± 5026.93	25666.67 ± 5877.15	23450 ± 726.29
Hematocrit (%)	47.67 ± 2.08	48.67 ± 5.51	47.33 ± 2.31	48 ± 2.65	48.33 ± 2.08
AF (%)	19.67 ± 4.04	21.67 ± 2.08	20.67 ± 3.51	18.67 ± 3.06	17.67 ± 3.51
ARB (405 nm)	0.35 ± 0.02^ab^	0.36 ± 0.02^ab^	0.38 ± 0.01^b^	0.38 ± 0.02^ab^	0.33 ± 0.01^a^
AL (405 nm)	26.67 ± 4.51^ab^	19.33 ± 2.31^a^	22 ± 0^a^	32.67 ± 6.66^b^	29.67 ± 2.31^ab^

SR: Survival rate, WG: Weight gain, FCR: Feed conversion ratio, SGR: Specific growth rate, TL: Total leukocyte, AF: Phagocytic activity, ARB: Respiratory burst activity, AL: Lysozyme activity. a, b, c, d: The numbers followed by different letters in the same column mean significantly different (p < 0.05) between treatments.

The SR values in tilapia following the challenge test with *S. agalactiae* bacteria revealed significant differences among the various treatments compared with the control without probiotic addition with the challenge test, as presented in Table-5. In addition, feed supplemented with *Bacterium* spp. supported the non-specific immune system. Specifically, *Bacterium* spp. treatment exhibited a notably higher SR value after the challenge test than the other treatments. Notably, the control fish which was not injected with *S. agalactiae*, consistently maintained a 100% SR throughout the study period.

**Table-5:** The performance of hematology and non-specific immune system of tilapia after being given the experimental feed and after the challenge test period.

Parameters	Treatments				

	*L. garvieae*	*P. megaterium*	*Bacterium* spp.	Control without probiotic addition, with challenge test	Control without probiotic addition , without a challenge test
SR (%)	76.67 ± 5.77^bc^	63.33 ± 2.89^ab^	86.67 ± 2.89^cd^	51.67 ± 16.07^a^	100 ± 0^d^
TL (sel.mm)^(-3)	16200 ± 3882.98	14500 ± 2528.34	15450 ± 1575.6	10366.67 ± 2194.5	12633.33 ± 2013.91
Hematocrit (%)	51 ± 1.73	51.33 ± 6.35	48.33 ± 5.77	53.33 ± 2.89	47.33 ± 2.52
AF (%)	28.67 ± 2.08^b^	29.67 ± 3.51^b^	28.67 ± 1.53^b^	20 ± 1^a^	18.67 ± 1.53^a^
ARB (405 nm)	0.57 ± 0.09^ab^	0.46 ± 0.02^a^	0.6 ± 0.14^ab^	0.77 ± 0.1^b^	0.68 ± 0.04^ab^
AL (405 nm)	163 ± 3.61	154 ± 3.61	162.33 ± 27.3	131 ± 26.96	125 ± 12.17

SR: Survival rate, TL: Total leukocyte, AF: Phagocytic activity, ARB: Respiratory burst activity, AL: Lysozyme activity. a, b, c, d: The numbers followed by different letters in the same column mean significantly different (p < 0.05) between treatments.

## Discussion

Six bacterial isolates, namely, *L. garvieae*, *P. megaterium*, *Bacterium*, *B. megaterium*, *B. subtilis*, and *B. pumilus*, previously identified for their capacity to produce digestive enzymes such as protease, lipase, amylase, and cellulose, were used in the present study. The antibacterial activity, hemolytic patterns, and detection of antibiotic resistance genes of these isolates were explored in this study, which had not previously been examined. Based on their hemolytic pattern γ and negative detection of antibiotic resistance genes, three isolates, *L. garvieae, P. megaterium*, and *Bacterium* spp., were selected as potential probiotic candidates (Table-3). While *L. garvieae, B. pumilus, Bacterium*, and *B. subtilis* inhabited *S. agalactiae* growth, *B. subtilis* exhibited *tetA* antibiotic resistance. Although *P. megaterium* lacked the ability to inhibit the growth of *S. agalactiae* bacteria, it demonstrated the ability to produce digestive enzymes [70], exhibited a g hemolytic pattern, and showed no detection of antibiotic resistance genes. In addition, *B. pumilus* displayed a b hemolytic pattern, indicating its potential as a pathogen for the host. Isolates that tested positive for antibiotic-resistance genes were also deemed unsafe for use as probiotic candidates. For this study, antibiotic resistance genes from the tetracycline group (*tetA*, *tetB*, *tetE*, *tetD*, *tetO*, and *tetQ*), the macrolide group (*ermB*), and the fluoroquinolone group (*qnrS*) were examined because these, three groups of antibiotics are commonly used for fish treatment in Indonesia [78]. Consequently, probiotic candidate isolates that tested for antibiotic resistance genes were excluded and not subjected to further *in vivo* testing on tilapia.

Extensive research has been conducted on antibiotic-resistance genes in bacteria used in aquaculture. Antibiotic resistance in bacteria raises concerns regarding their suitability as probiotic candidates because they serve as reservoirs of antibiotic-resistance genes. Probiotics consisting of bacteria devoid of antibiotic resistance genes offer enhanced animal and environment safety, thereby contributing to improved food safety [79]. Anokyewaa *et al*. [80] identified a commercial probiotic product in China that contains live bacteria and is antibiotic-resistant, posing potential risks to the food safety of aquaculture products. The administration of probiotics containing live bacteria with resistance genes raises significant concerns about the health of fish and the environment. Moreover, resistance genes can be transferred between bacteria, causing non-exposed bacteria to become antibiotic-resistant [81]. A previous study has shown that resistance genes can be transferred from Gram-positive to Gram-negative bacteria [82]. In addition, Gram-negative bacteria can transfer resistance genes to other Gram-negative bacteria [83]. Dissemination of resistance genes in aquaculture has been observed in fish and their environment, including pond sediments [84–86], and the presence of resistance genes in pathogenic bacteria in fish has been reported to exacerbate the condition of aquaculture and the fish environment [87]. In addition, there is a potential ineffective use of antibiotics for fish treatment. This study provides new insights by producing probiotic bacterial isolates that do not detect specific antibiotic-resistance genes. The food chain can transmit antimicrobial-resistant bacteria and resistance genes from aquatic organisms to their environment [88, 89]. Bacteria play a crucial role as hosts for antibiotic resistance genes and indirectly influence mobile genetics elements (MGEs) [90]. The transfer of resistance genes among bacteria through MGEs is plausible, particularly from probiotic bacteria that contain antibiotic resistance genes. Mobile genetic elements carrying resistance genes are linked to humans and aquaculture environments [91, 92]. Antibiotic-resistant bacteria present in fish farming waste pose potential threats to environmental health [93]. On the basis of this comprehensive review, *L. garviae*, *Bacterium*, and *P. megaterium* have been identified as more qualified and safe probiotic candidates. As a result, the efficacy of these isolates has been further assessed using tilapia as the test organism.

Bacillus spp. is renowned for its ability to produce natural antimicrobial lipopeptides that effectively inhibit the growth of pathogenic bacteria [94, 95]. In addition, antiviral, antimycoplasma, and antiprotozoal activities have been demonstrated [96]. In previous studies, the bacterium was identified as a producer of essential enzymes such as protease, lipase, amylase, and cellulose. Furthermore, it inhibits the growth of *S. agalactiae bacteria*. The *in vivo* evaluation of tilapia revealed promising results when *L. garvieae, P. megaterium*, and *Bacterium* were supplemented, as they significantly enhanced tilapia growth compared with the control group (Table-4). According to Ghosh *et al*. [97], the administration of probiotics at concentrations ranging from 10^6^ cells/g to 10^8^ cells/g resulted in favorable growth outcomes and improved reproductive performance in fish. Probiotic bacteria residing in the digestive tract play a key role in synthesizing essential nutrients, such as proteins and essential fatty acids, while producing enzymes that facilitate digestion [98]. The increased growth observed in fish supplemented with probiotics can be attributed to the higher presence of probiotic bacteria in the digestive tract, which aids in nutrient decomposition and provides additional enzymes, vitamins, and amino acids for fish growth [99, 100]. The post-challenge SR values of *S. agalactiae*-exposed fish exhibited a significant correlation with the nonspecific immunological system parameter values compared with the control fish (Table-5). This study highlights that probiotic supplementation in tilapia enhances their resistance to *S. agalactiae* infection, as evidenced by the higher SR values observed in the probiotic-treated tilapia after the challenge test compared with the control group. The role of probiotics as bio-growth supplements and promoters of disease resistance has been well documented in previous studies. The innate immune system in fish consists of both cellular and humoral immune systems. The cellular immune system consists of macrophages, monocytes, and granulocytes, whereas the humor is composed of lysozyme, immunoglobulin, and the complement system. The immune system is the body’s initial defense mechanism against invading pathogens [101, 102]. Among the cellular immune components, leukocytes are crucial in protecting the body against diseases [103].

Probiotics stimulate lymphoid tissue associated with the intestines of fish, triggering an immune response [104]. The cellular immune response involves both innate and adaptive cells, including leukocytes and macrophages, which play a significant role in cellular defense mechanisms against pathogens [105]. Physical, humoral, and cellular factors influence the innate immune response in fish. In the present study, we also observed a significant increase in the hematocrit percentage of fish subjected to stressors [106]. Phagocytic activity is an important line of defense in which serum contains various peptides, including lysozyme, antibodies, complement factors, and other lytic factors. These components prevent the adherence and colonization of microorganisms, thus contributing to the prevention of infections and diseases [107]. Probiotic supplementation enhances non-specific immune system and improves blood parameters [108]. Lysozyme, an integral part of the innate immune system in fish, exhibits lytic activity against pathogenic bacteria and demonstrates varying activity based on factors such as size, age, water temperature, pH, toxicity, infection, and stress level [109]. Lysozyme activity is crucial to inhibit the growth and invasion of pathogens in fish [110].

## Conclusion

This study concludes that *Lactococcus garvieae*, *Priestia megaterium*, and *Bacterium* spp. fulfilled the requirements to be considered probiotic candidates. These candidates have antibacterial properties, do not have the potential to become pathogens, and do not contain antibiotic-resistant genes. *Bacterium* and *L. garvieae* supplemented in tilapia feed demonstrated improved growth, hematological parameters, non-specific immune system parameters, and increased resistance to *S. agalactiae* infection in tilapia.

## Authors’ Contributions

MM and AI: Designed the study and validation. MM, AI, and IWTW: Methodology. AI and AML: Data collection and data curation. MM, AI, IWTW, and AML: Formal analysis. AI, IWTW, and AML: Investigation and review and editing. MM, AI, and AML: Writing-original draft preparation. All authors have read, reviewed, and approved the final manuscript.
